# Sleep apnea syndrome: experience of the pulmonology department in Ibn Sina Hospital, Rabat, Morocco

**Published:** 2012-10-10

**Authors:** Asmaa Jniene, Mustapha el Ftouh, Mohamed Tawfiq el Fassy Fihry

**Affiliations:** 1 Department of pulmonology, Ibn Sina hospital, Rabat, Morocco

**Keywords:** Obstructive sleep apnea hypopnea syndrome, characteristics, management, reliability, Berlin questionnaire, Epworth sleepiness scale

## Abstract

**Introduction:**

Sleep apnea syndrome is a highly prevalent disorder that is still underdiagnosed and undertreated and whose obstructive form is the most common. The diagnosis is suspected on clinical signs collected by interrogation and questionnaires (Berlin questionnaire and Epworth sleepiness scale), then confirmed by objective sleep study findings (polygraphy or polysomnography). It is necessary to conduct studies in each context on the characteristics and management of sleep apnea syndrome comprising the testing of reliability of the questionnaires.

**Methods:**

Prospective and descriptive study of 104 patients addressed to sleep consultation at pulmononology Department of Ibn Sina Hospital, Morocco over a period of 5 years (January 2006 to December 2010), agreed to participate in the study, responded to a predetermined questionnaire, and benefited from clinical examination and paraclinical tests including a polygraphy or a polysomnography

**Results:**

59(56.7%) patients had an obstructive sleep apnea-hypopnea syndrome with a similar prevalence in both sexes. 32.2% of patients were obese and 28,8% had cardio-vascular diseases. Snoring, excessive daytime sleepiness and witnessed apnea were found in respectively 79.7%, 50.8% and 16.9%. Berlin questionnaire and Epworth sleepiness scale had an acceptable internal consistency against apnea hypopnea index with a Cronbach’s alpha coefficient respectively 0.79 and 0.78. Depending on severity, clinical impact and results of investigations, the adequate treatment has been proposed based on the 2010 recommendations for clinical practice.

**Conclusion:**

This study has provided an idea about the profile and the management of patients having an obstructive sleep apnea-hypopnea syndrome and showed that both Berlin questionnaire and Epworth sleepiness scale are two simple and reliable methods in our context. A larger and further study across the country should be considered.

## Introduction

The sleep apnea syndrome (SAS) is a highly prevalent disorder occurring in at least 4% of males and 2% of females [1–3], which results in periods of recurrent reduced breathing (hypopnea) or periods of breathing cessation (apnea). The most common form of sleep apnea is called obstructive sleep apnea-hypopnea syndrome (OSAHS) and is caused by the partial or complete collapse of the upper airway during sleep that leads to oxyhemoglobin desaturation and terminate by brief microarousals causing sleep fragmentation and alteration of its quality [4].

Patients habitually present with usual loud snoring, witnessed apnea and excessive daytime sleepiness (EDS). The diagnosis is based on combined assessment of clinical features with objective sleep study findings by polygraphy or polysomnography [5].

Untreated OSAHS increases the risk for car accidents [6], and worsens quality of life and mood [7]. However, the major health risk is represented by cardiovascular events whether acute (stroke, myocardial infarction and nocturnal sudden death) or chronic (systemic hypertension, coronary artery disease and heart failure) [8]. On the other hand, patients with OSAHS have a high prevalence of other cardiovascular risk factors that is obesity, hyperlipidemia, and diabetes (metabolic syndrome) [9].

Since the original description by Sullivan and coworkers in 1983 of continuous positive airway pressure (CPAP) treatment, it remains the mainstay of treatment for moderate to severe OSAHS in adults [10–13]. Effective treatment of the disorder has been associated with major improvements in quality of life and also a diminished risk of cardiovascular morbidity and mortality [14].

Despite the growing importance due to its high prevalence, its social and health repercussions, and its clear link to cardiovascular disease, the OSAHS is still frequently underdiagnosed and undertreated that’s why epidemiological studies should be done in each population and context in order to emphasize the recognition of the disease, to describe its characteristics and management, also to test the reliability of its principal questionnaires (Berlin questionnaire and the Epworth sleepiness scale ESS) which constitute an initial step in the diagnosis since they may prioritize patients for polygraphy or polysomnography depending on their results.

## Methods

### Study population

This study was a prospective and descriptive analysis of records of 104 patients complaining of sleep disorders and addressed to the sleep consultation in the pulmonology department of Ibn Sina Hospital in Rabat (Morocco) over a period of 5 years (January 2006 to December 2010). Oral consent was obtained from the all patients for inclusion in the study. Authorization to conduct the study was provided by the hospital authorities.

Detailed information was recorded by a pulmonologist

**Clinical information:** Provenance and epidemiological characteristics (age, sex, medical coverage); clinical history, median duration between the onset of symptoms and consultation, research of diurnal (daytime sleepiness, fatigue on awakening, headache, memory loss, decreased concentration, depression, irritability) and nocturnal symptoms (snoring, witnessed apnea, fragmented Sleep, nocturia, sweating); estimated probability of clinical sleep disorders by Berlin questionnaire which consists of 3 categories related to the risk of having sleep apnea. When there are at least 2 positive categories it is in favor of a strong probability of the disease; Epworth Sleepiness Scale which is a scale intended to measure daytime sleepiness by use of a very short questionnaire that asks the subject to rate the probability of falling asleep on a scale of increasing probability from 0 to 3 for eight different situations. The scores are added together to obtain a single number. A score above 10 is considered in favor of EDS which can be associated to sleep disorders.

A complete clinical examination including a specialized ear, nose and throat examination (ENT) and also the calculation of body mass index (BMI) by the weight (kg) / size (m^2^). The World Health Organization defines overweight as a BMI equal to or greater than 25 kg/m^2^ and obesity as a BMI equal to or greater than 30 kg/m^2^.

**Paraclinical examination**: All the patients underwent a chest X-ray, spirometry and electrocardiogram. The patients also had a polygraphy or polysomnography according to the clinical probability of SAS which was based on the interrogation (presence and / or frequency of occurrence of the main signs suggestive of the SAS), the ESS and the Berlin questionnaire. When the probability was high or low and in the absence of neurologic signs, the patients had underwent polygraphy. When the diagnosis was uncertain or when the probability was medium or in the presence of neurologic signs, the polysomnography was performed.

Standard overnight polygraphy included airflow that was measured by a nasal pressure transducer, respiratory effort by thoracoabdominal belts, and arterial oxyhemoglobin saturation by a pulse oximeter placed on the patient’s finger. Polysomnography included in addition to these measurements, the recordings of electroencephalogram, electro-oculogram, submental myogram, and electrocardiogram.

Apnea was defined as the absence of the airflow over 10 seconds. An obstructive apnea was defined as the absence of airflow in the presence of rib cage and/or abdominal movements, and a central apnea was defined as the absence of both airflow and rib cage and abdominal movements. Events were scored as hypopneas when there was a decrease of at least 50% of a validated flow signal from the baseline or decrease below 50% or appearance of inspiratory plateau associated with a transcutaneous desaturation of at least 3% or when an electroencephalogram microarousal occurred [15].

Both apnea and hypopnea are added and expressed as an index: apnea-hypopnea index (AHI) which is used to assess the severity of sleep apnea based on the total number of apneas and hypopneas per hour. The severity is classified mild when the AHI was ranged from 5 to 15/h, moderate when the range was 15 to 30, and severe when it was above 30. Depending on the severity, the clinical impact and the results of investigations, the adequate treatment has been proposed based on the 2010 recommendations for clinical practice [15].

### Expression of results

Data entry and analysis was done using SPSS 17.0 for Windows^®^ (SPSS Inc, Chicago, IL, USA). Data were expressed as mean ± standard deviation and range, and for data with skewed distribution as median and range. Relationships among continuous quantitative variables were evaluated using student’s t test and continuous qualitative variables by chi square test. Correlations between the ESS and AHI, and between Berlin questionnaire and AHI were determined by linear regression. Cronbach′s Alpha Coefficient measured the internal consistency. Coefficients above 0.7 are generally regarded as acceptable, 0.8 and above are good, and 0.9 and above are considered excellent. Differences were considered statistically significant if p was less than 0.05.

## Results

### Epidemiology

During the 5 years study period, a total of 104 patients were enrolled. The mean age of patients was 47.5 years ± 12.4 with a sex ratio 1/1. The mean age of patients with OSAHS was 47.4 years ± 13 and 29 (49%) of the patients were female among whom 15 (51.7%) were postmenopausal. 44 patients (42.3%) had no medical coverage among whom 21 (35.6%) patients had OSAHS.

### Clinical findings

The patients’ antecedents are represented by Table 1. The most reported diurnal and nocturnal signs are represented by Table 2. **Berlin questionnaire:** It was positive in favor of a strong probability of SAS in 50% of patients among whom 67.8% of the patients had OSAHS (p: 0.03). Berlin questionnaire had an acceptable internal consistency against apnea hypopnea index with a Cronbach’s alpha coefficient at 0.79. **Epworth Sleepiness Scale:** Averaged 10.1 ± 5.4 (range: 1-21). It was over 10 in favor of EDS in 60 subjects (57.7%) among whom 81.3% subjects had OSAHS (p: 0,04). ESS had an acceptable internal consistency against apnea hypopnea index with a cronbach’s alpha coefficient at 0.78. Data from the clinical examination are shown in Table 3.

**Table 1:** The patients’ past medical history of patients with obstructive sleep apnea-hypopnea syndrome seen at the pulmonology department of Ibn Sina Hospital, Rabat, Morocco from January 2006 to December 2010

	Number ^a^(%)	Number ^b^(%)
**Cardiovascular**		
Hypertension	28 (26,9)	17 (28,8)
Ventricular extrasystole	2 (1,9)	1 (1,7)
Arrhythmia	3 (2,9)	2 (3,4)
**Endocrine**		
Cushing	1 (1)	0
Thyroid nodule	3 (2,9)	2 (3,4)
Acromegaly	11 (10,6)	6 (10,2)
Goiter	6 (5,8)	5 (5,8)
Hypothyroidism	2 (1,9)	2 (3,4)
Diabetes	22 (21,2)	9 (15,3)
Hyperlipemia	2 (1,9)	1 (1,7)
**Pulmonary**		
Asthma	4 (3,9)	1 (1,7)
Pulmonary fibrosis	1 (1)	0
Smoking	15 (14,4)	12 (20,3)
**ENT**		
Allergic rhinitis	14 (13,5)	9 (15,3)
Chronic sinusitis	4 (3,9)	1 (1,7)
Tonsillectomy	3 (2,9)	1 (1,7)
Uvulo-palato-pharyngoplasty	1 (1)	0
**Neuropsychiatric**		
Epilepsy	1 (1)	0
Chronic migraine	2 (1,9)	1 (1,7)
Chronic depression	2 (1,9)	1 (1,7)
**Nephrological**		
Renal failure	2 (1,9)	2 (3,4)
**Gastrointestinal**		
gastroesophageal reflux	8 (7,7)	2 (3,4)
**Dysmorphic craniofacial defects**		
Mac cune Albright syndrom	1 (1)	1 (1,7)
Craniosynostosis in Apert type with Arnold Chiari malformation type 1	1 (1)	1 (1,7)
Achondrodysplasy	1 (1)	1 (1,7)
**Highway accidents**	**3 (2,9)**	**1 (1,7)**

a all subjects, n = 104

b patients with obstructive sleep apnea-hypopnea syndrome, n= 59

**Table 2:** The most reported clinical signs in patients with obstructive sleep apnea-hypopnea syndrome seen at the pulmonology department of Ibn Sina Hospital, Rabat, Morocco from January 2006 to December 2010

Clinical signs	Number ^a^(%)	Number ^b^ (%)
**Diurnal signs**		
Daytime sleepiness	53 (51)	30 (50,8)
fatigue on awakening	35 (33,7)	20 (33,9)
Headache	26 (25)	18 (30,5)
Irritability	25 (24)	14 (23,7)
Decreased concentration	23 (22,1)	12 (20,3)
Memory Loss	17 (16,3)	9 (15,3)
Depression	19 (18,3)	3 (5,1)
**Nocturnal signs**		
Snoring	77 (74)	47 (79,7)
Witnessed Apnea	39 (37,5)	10 (16,9)
Fragmented Sleep	15 (14,4)	12 (20,3)
sweating	14 (13,5)	9 (15,3)
Nocturia	10 (9,6)	5 (8,5)

a all subjects, n = 104

b patients with obstructive sleep apnea-hypopnea syndrome, n= 59

**Table 3:** Data from the clinical examination of of patients with obstructive sleep apnea-hypopnea syndrome seen at the pulmonology department of Ibn Sina Hospital, Rabat, Morocco from January 2006 to December 2010

Clinical examination	Number ^a^(%)	Number ^b^(%)
BMI (mean kg/m^2^ ± standard deviation)	30,5 ± 6,9	30,6 ± 7,6
**Pulmonary examination**		
Normal	103 (99)	59 (100)
Abnormal (pulmonary fibrosis)	1 (1)	0
**ENT examination**		
Normal	84 (80,8)	47 (79,7)
Abnormal	20 (19,2)	12 (20,3)
Big tonsils	5 (4,8)	5 (5,8)
Dysmorphic syndrome with reduced antero-posterior pharynx	5 (4,8)	4 (6,8)
Macroglossia	5 (4,8)	4 (6,8)
Nasal septum deviation	6 (5,8)	2 (3,4)
Long soft palate	4 (3,8)	2 (3,4)
Oropharynx with small soft palate located below	2 (1,9)	1 (1,7)
Velar hypertrophy with thickening of the posterior pillars	1 (1)	1 (1,7)
Retrognathia, short neck and hypoplasia of the dentition of the lower jaw	1 (1)	1 (1,7)
**Cardiovascular examination**		
Hypertension	6 (5,8)	4 (6,8)
**Examination of the thyroid gland**		
Goiter	4 (3,9)	2 (3,4)
**Neurological examination**		
Normal	104 (100)	0
Abnormal	0	0

a all subjects, n = 104

b patients with obstructive sleep apnea-hypopnea syndrome, n= 59

### Paraclinical examination

Data from the paraclinical examination are shown in Table 4. Polygraphy was performed in 64 subjects (61.5%), whereas the polysomnography was perfomed in 40 subjects (38.5%). 59 (56.7%) patients had an OSAHS among wich 19 was mild, 20 moderate and 20 severe. No positional OSAHS nor central apnea syndrom were diagnosed. Among the severe OSAHS, 1 case of obesity-hypoventilation syndrome associated was recorded.

**Table 4:** data from the paraclinical examination of patients with obstructive sleep apnea-hypopnea syndrome seen at the pulmonology department of Ibn Sina Hospital, Rabat, Morocco from January 2006 to December 2010

Paraclinical findings	Number ^a^(%)	Number ^b^ (%)
**Chest X ray**		
Normal	98 (94,2)	57 (96,6)
Abnormal	6 (5,8)	3 (5,1)
Cardiomegaly	2 (1,9)	1 (1,7)
Enlargement of the superior mediastinum	3 (2,9)	1 (1,7)
Interstitial Syndrome	1 (1)	0
**Spirometry**		
Normal	94 (90,4)	56
Restrictive profile	7 (6,7)	1 (1,7)
Obstructive ventilatory defect	3 (2,9)	2 (3,4)
**Electrocardiogram**		
Normal	92 (88,5)	54
Abnormal	12 (11,5)	5 (8,5)
Right atrial hypertrophy	10 (9,6)	4 (6,8)
Arrhythmia	2 (1,9)	1 (1,7)

a all subjects, n = 104

b patients with obstructive sleep apnea-hypopnea syndrome, n= 59

### Treatment

Depending on the severity, the clinical impact and results of investigations, therapeutic approaches have been proposed: All overweight patients whose BMI exceeded 25 Kg /m^2^ received dietary guidelines and were referred to a dietician whether or not they had a sleep-related breathing disorder. 5 patients were readressed to ENT consultation to receive a tonsillectomy before considering a mandibular advancement device or a CPAP. No indication for surgery was retained. Mild OSAHS received dietary guidelines. Moderate OSAHS received CPAP or mandibular advancement device as first-line, these were sent to a specialized dentist. As concern severe OSAHS, they had received CPAP as first-line and were sent to a specialized service provider. The patient with the association of OSAHS and obesity hypoventilation syndrome had beneficed of a non-invasive ventilation initially. Patients with asthma and / or associated with allergic rhinitis had received specific treatment for their respiratory disease. Patients with gastroesophageal reflux disease had received inhibitors of proton pump. All patients with OSAHS had benefited from therapeutic education in order to emphasize about the benefits and the correct compliance to treatment.All the patients were readressed to their home consultations for monitoring and receiving specific treatment of their primary pathology (consultation of Cardiology, ENT, endocrinology, neurology, nephrology and psychiatry).

### Evolution

Was marked by the low number of patients returning for follow-up consultation: Only 9 (15.3%) patients with OSAHS had benefitied of a control by polygraphy among whom 7 were treated by CPAP, one by non invasive ventilation and one by mandibular advancement device. The control had objectified a normalization of the AHI, and also the quality of life and a reduction in daytime sleepiness objectified by the normalization of the ESS. The other patients were lost to sight.

## Discussion

The SAS is a new disease that has been individualized until 1976 by Guilleminault et al. [16]. Among the well recognized risk factors in the development of the disease, the male gender is well known where the risk is two to three times higher in the general population. This increased risk may be related to differences in the distribution of adipose tissue in men which have a central repository fat mainly around the neck, trunk and abdominal viscera comparing to women. Otherwise, preliminary data from the study cohort of Wisconsin (541 women aged 30 to 60 years) showed that menopause seems to be a risk factor for OSAHS and hormone therapy has a protective action [17].

In adults, the prevalence of OSAHS also increases with age. In the study of Ancoli-Israel et al. which included 427 subjects over 65 years, the frequency of an AHI higher than 10 was 70% for men and 56% for women, that was 3 times higher comparing to middle-aged adults [18]. Duran et al. have made similar findings [19]. Overweight and especially obesity is also a risk factor for conventional SAHOS. Indeed, mild to moderate obesity was associated with a significantly increased prevalence of sleep apnea [20] with an average body mass index of 30-35 kg/m2 in most recent series of literature [3, 21].

Our study involved a population suspected sleep apnea syndrome addressed in sleep consultation, there was no sex predominance, among women 51,7% were postmenopausal, the average age of patients was 47,4 years ± 1 and the mean BMI was 30,6 kg/m^2^ ±7,6.

Currently recognized and considered as a real public health problem, the SAHOS, in addition to its frequency, leads to an increase of cardiovascular, endocrine and psychic morbidity and mortality in addition to the significant increased risk of occupational and highway accidents. Indeed, there is an independent risk for the development of hypertension especially resistant to treatment, also coronary artery disease, heart failure and stroke [22–31]. It has been observed in patients with severe untreated OSAHS with CPAP, a significantly increased cardiovascular morbidity and mortality compared to controls, whereas those treated with CPAP, it is comparable to those of the general population [32].

In our study 17 (28,8%) patients with OSAHS had cardiovascular antecedents represented mainly by hypertension. Otherwise SAHOS increases by 40% the risk of developing the metabolic syndrome, which is in itself a cardiovascular risk factor [33–35]. Also, acromegaly and hypothyroidism represent the most classically endocrine disorders associated to OSAHS related to macroglossia encountered in these diseases and craniofacial abnormalities associated to acromegaly. Other conditions such as Cushing′s syndrome have been described in association with the disease [35].

In our study, among subjects with OSAHS 9 (15,3%) were diabetics, 6 (10,2%) had acromegaly, 2 (3,4%) had hypothyroidism and 1 (1,7%) was treated for hyperlipidemia.

EDS lead to psychological consequences represented by significant deficiencies in the quality of life, cognitive performance, and social functioning [7, 36, 37]. 14 (23,7%) of our patients had irritability from whom 2 reported a chronic depression under treatment.EDS is also the source of accidentological risk where the relative risk among patients with SAS compared to the general population is increased by a factor of 3 to 7. 1 patient reported a serious car accident because of his sleep while driving.

Under CPAP treatment, the disappearance of this EDS is associated to a significant improvement in quality of life [38, 39] and a very significant reduction in accidents. A study showed that current smokers had a risk three times greater of developing OSAHS than subjects who never smoked, but these results were not confirmed and smoking cannot be considered as an established risk factor for OSAHS. Appropriate epidemiological studies are needed [40]. In our study, 12 (20,3%) of patients with OSAHS were smokers. Rhinitis is a risk factor for developing an OSAHS because of the local inflammation and congestion. 9 (15,3%) of patients with OSAHS had rhinitis in our study. Chronic renal failure is associated with the SAS but the mechanisms underlying this association are still unclear [41]. In our study, 2 (3,4%) patients with OSAHS was at the terminal stage of chronic renal failure. Finally, during OSAHS, swallowing reflex is impaired. A relationship between apnea and gastroesophageal reflux disease (GERD) is found. The number of episodes of reflux is reduced by positive airway pressure and number of awakenings on apnea is reduced by anti-H_2_ [42]. In our study 2 (3,4%) had GERD.

The most common clinical signs in the SAHOS are represented by EDS, snoring and witnessed apnea. When examining a patient suspected of the disease, it is highly recommended to ask the partner who can often provide important additional information based on direct observation of the patient during his sleep [3, 43].

Although sleep apnea is the most common cause of EDS, this taken alone cannot be a criterion for discrimination of clinical disorder, indeed between 30 and 50% of the general population report a significant sleepiness because study participants often confuse fatigue and EDS. On the other hand, several studies have shown that the severity of sleep apnea and EDS did not match [44] since many other sleep disorders can also cause EDS.

Snoring is a very common sign in the general population whose prevalence is increasing in both sexes after 35 years. It is assumed that 60% of men and 40% of women aged 40 to 60 are habitual snorers. That is why taken alone, snoring is not considered synonymous to SAHOS but when it is regularly interrupted by apneas and it reappears in an intense way for the resumption of breathing it is very suggestive of the disease [45].

As concern apnea, it should be noted that it is a good predictor sign but does not predict the severity of the disease [45]. Fragmented sleep is frequently reported by patients, it reflects the effect of recurrent arousals. In our study, 47 (79,7%) patients reported snoring 10 (16,9%) a witnessed apnea, and 12 (20,3%) a fragmented sleep.

The severity of EDS can be evaluated subjectively by various questionnaires; the most widely used is the ESS [44]. It was introduced in 1991 by Dr Murray Johns of Epworth Hospital in Melbourne, Australia. The Berlin questionnaire is an instrument validated to use in the western population to determine the occurrence of risk factors for OSA, namely snoring behavior, wake-time sleepiness or fatigue, and the presence of obesity or hypertension. The predictive performance of the Berlin questionnaire has been evaluated in the population of Cleveland, Ohio, with a sensitivity of 86% and specificity of 77% [46].

In the present study, the reliability of these two questionnaires was tested and showed that they are two simple and reliable methods for measuring daytime sleepiness and identifying patients who are likely to have sleep apnea.

On physical examination, and more specifically the ENT examination, the most frequently encountered abnormality is the oropharyngeal narrowing with or without an increase in deposits of soft tissues [47]. This was the most frequently found in the study in addition to macroglossia and big tonsils.

Polysomnography is considered the gold standard for the diagnosis of OSAHS. However, the access is restricted due to its high cost, the need for continuous attention, and a considerable investment of time on the part of medical staff. In the present study we performed polysomnography in patients when the diagnosis was uncertain or when the probability was medium or in the presence of neurologic signs. Otherwise, the polygrapy was performed. The indication for treatment was based on the 2010 french recommandations for clinical practice [15].

## Conclusion

The present study has given a brief idea on the characteristics, the management of patients who suffer from OSAHS and also shown an acceptable reliability of the Berlin questionnaire and the ESS in this sample of Moroccan population. Nevertheless, there are limitations. First it only shows the characteristics of OSAHS in a group of subjects being aware of their condition health, living mainly in big cities of Morocco. Second it is limited by the low number of patients’ recruitment that may be due to sleep medicine which is still in a stage of development in Morocco and SAS is frequently under-diagnosed and still underestimated not only by the public but also by the medical community. In the other hand it may be explained by the difficulty to access to diagnosis (examination cost for subjects with no Medical coverage). And finally the number of patients returning for follow-up consultation was very low. A larger study across the country should be considered.
